# Non-survival rat endovascular testbed for early-stage evaluation of untethered magnetic microrobots

**DOI:** 10.1016/j.mex.2026.103906

**Published:** 2026-04-09

**Authors:** Ping Wang, Tuan-Anh Le, Prabh Singh, Hassan Albadawi, Husnu H. Alabay, Hakan Ceylan

**Affiliations:** a Department of Physiology and Biomedical Engineering, Mayo Clinic, Scottsdale, AZ, USA; b Department of Family Medicine, Conemaugh Memorial Medical Center, Johnstown, PA, USA; c Division of Vascular and Interventional Radiology, Mayo Clinic, Scottsdale, AZ, USA

**Keywords:** Microrobots, Millirobots, Medical robots, Endovascular robots, Endovascular navigation, Rat inferior vena cava

## Abstract

Early-stage in vivo evaluation of microrobot navigation controllability and acute device-tissue interaction remains methodologically underdeveloped. In vitro and ex vivo approaches often fail to balance technological readiness, physiologic relevance, and reproducibility. To address this gap, here we present a non-survival, acute in vivo endovascular testbed in the rat inferior vena cava (IVC). Rat IVC supports studying microrobots with characteristic sizes of 0.1–4 mm and integrates key methodological components, including vascular access, continuous blood flow, anticoagulation strategies, fluoroscopic imaging guidance, and acute in vivo performance benchmarking. The testbed enables systematic assessment of minimum viable in vivo performance metrics for early-stage endovascular microrobots, including image-guided navigation success rate, traversal time, path deviation, acute vessel interaction, deployment/retrieval and device integrity. Common failure modes and practical troubleshooting strategies are provided to support reproducibility across laboratories. The non-survival design is a deliberate methodological choice that isolates acute, engineering-relevant performance while avoiding confounding long-term biological responses. Accordingly, the protocol addresses questions of early design feasibility and control performance but does not evaluate chronic biocompatibility or therapeutic efficacy. By establishing a reproducible intermediate validation stage, this testbed de-risks subsequent survival and large-animal studies in translational microrobot development.

## Highlight

• Enables quantitative assessment of early-stage in vivo performance metrics, including navigation success rate, traversal time, path deviation, acute vessel interaction, and device integrity.
• Provides an intermediate validation stage that de-risks subsequent survival and large-animal studies for translational microrobot development.
• Troubleshoots common failure modes and provides practical strategies to enhance cross-laboratory reproducibility.

## Graphical abstract

**Figure:**
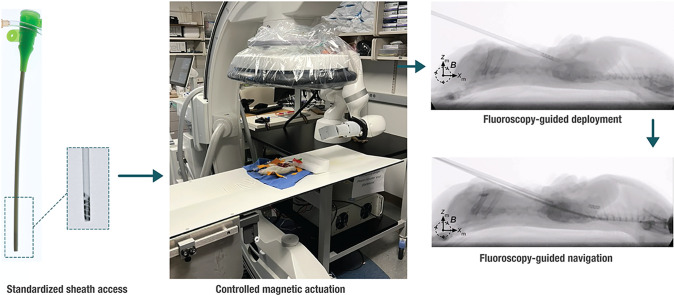

## Specifications table

**Table:** 

**Subject area**	Engineering
**More specific subject area**	Medical robotics; Microrobotics; endovascular microrobots
**Name of your method**	Non-survival endovascular testbed for microrobot
**Name and reference of original method**	Tuan-Anh Le, Husnu Halid Alabay, Prabh Singh, Melissa Gioia Austin, Sanjay Misra, Hakan Ceylan. Physiologically Adaptive Soft Millirobot for Atraumatic Endovascular Therapy. *Advanced Functional Materials*, 2026. DOI: 10.1002/adfm.202531400
**Resource availability**	N/A

## Background

Microrobots are being actively explored for endovascular navigation, sensing, and treatment across a wide range of vascular diseases, including ischemic stroke, aneurysm, and stenosis [[1], [2], [3], [4]]. Their untethered, remotely controlled operation enables access to complex and distal vascular regions beyond the reach of conventional catheter-based tools [[5], [6], [7]]. Recent advances in design, propulsion, and image-guided control have further improved navigation precision, supporting the emergence of translational endovascular microrobotics research [8,9].

To date, however, characterization of endovascular microrobots has been predominantly conducted in simplified experimental settings. Microfluidic channels and phantom vessels with well-defined geometries have enabled systematic evaluation of helical swimming, rolling, crawling and swarming locomotion under controlled flow conditions, including water, blood-mimicking fluids, and whole blood [2,9,10]. These platforms have been instrumental in exploring feasibility of fundamental robotics, refining propulsion efficiency, and demonstrating multi-functional capabilities, such as drug delivery and biodegradability. More recently, experiments in ex vivo vessels and complex flow loops have pushed realism further by introducing biological vascular geometries, branching, and blood rheology [4,11].

Nevertheless, fully replicating in vivo conditions remains a formidable challenge. In vitro and ex vivo models cannot capture critical factors present in living subjects, including pulsatile and autoregulated hemodynamics; respiratory- and cardiac-induced vessel motion; active vasoreactivity and dynamic changes in vessel tone; endothelial interactions and surface friction under physiologic lubrication; blood rheology with cellular components (e.g., hematocrit-dependent viscosity, cell-device interactions, and margination effects); coagulation and thrombogenic responses; immune and inflammatory interactions; and complex vessel wall mechanics, including compliance, anisotropy, and remodeling. In addition, in vivo environments impose systemic constraints such as organ-level coupling, toxicity, metabolic regulation, and temperature homeostasis, as well as practical limitations associated with real-time clinical imaging modalities (e.g., fluoroscopy, ultrasound) and restricted visualization. Microrobots that navigate reliably in benchtop settings may therefore behave unpredictably when exposed to this multiscale, dynamically coupled in vivo milieu. The lack of standardized, accessible in vivo testing paradigms, particularly within engineering-driven microrobotics research, has further limited reproducibility, cross-platform comparison and technological readiness level toward translation [12].

Acute in vivo evaluation in healthy animals represents a critical intermediate step between extensive in vitro refinement and longitudinal survival studies conducted in disease models prior followed by clinical trials [13,14]. While these acute studies are primarily intended to assess engineering-relevant performance, they also provide an essential opportunity to establish baseline biological safety. The vascular endothelium is a highly mechanosensitive and biologically active interface that regulates thrombosis, inflammation, vascular tone, and smooth muscle cell behavior [[15], [16], [17]]. Even brief mechanical perturbations can induce endothelial dysfunction, platelet activation, and pro-inflammatory signaling [15,[17], [18], [19]]. Accordingly, acute in vivo testing enables assessment of early safety markers such as endothelial integrity, thrombus formation, and gross vessel wall injury, which are foundational for subsequent translational progression. Despite rapid advances in microrobot design and control, endovascular microrobotics lacks a standardized, accessible acute in vivo framework for evaluating untethered devices [12]. Current studies remain largely confined to benchtop or ex vivo models without any physiologically relevant hemodynamic and imaging conditions to assess immediate vascular safety [17,20,21].

To bridge this gap, we present a reproducible, non-survival rat inferior vena cava (IVC) in vivo testbed that enables controlled assessment of microrobot navigation, image-guided control performance, and acute device-vessel interactions. IVC was chosen as the primary testbed vessel due to its mechanical, anatomical, and experimental characteristics that facilitate early-stage in vivo evaluation of endovascular microrobots. First, the IVC offers a relatively large luminal diameter (approximately 1.5–3.3 mm in rats), which enables reliable sheath insertion and microrobot deployment into human-scale-compatible vessels while minimizing the risk of complete occlusion. This configuration facilitates controlled navigation and retrieval, both of which are essential for reproducible performance assessment. Second, the IVC demonstrates continuous flow with reduced pulsatility and lower shear stress compared to arterial systems. This environment permits isolation of magnetic actuation and device-vessel interaction dynamics without significant pulsatile disturbances. Third, the IVC is anatomically accessible through midline laparotomy, which allows more trivial visualization and controlled exposure using multiple medical imaging modality. This accessibility supports precise catheter placement and consistent experimental geometry across subjects. Finally, the IVC models clinically relevant endovascular workflows, such as sheath-based access and fluoroscopic-guided navigation, thereby enhancing translational relevance.

Although this model captures above-mentioned key features relevant to endovascular navigation, it does not fully replicate the complexity of human vascular anatomy and physiology. In particular, anatomical depth and flow dynamics remain simplified. Human arteries exhibit strongly pulsatile flow, which is not represented in this venous flow model. However, due to its proximity to the heart, in this protocol, we observed periodic vascular motion, capturing an important aspect of arteries, which can be tested in this venous platform. Accordingly, this testbed serves as an intermediate validation stage, enabling controlled evaluation of navigation, device-vessel interactions, and imaging compatibility prior to progression to large-animal and human-scale systems.

### Method details

**Fig. 1:**
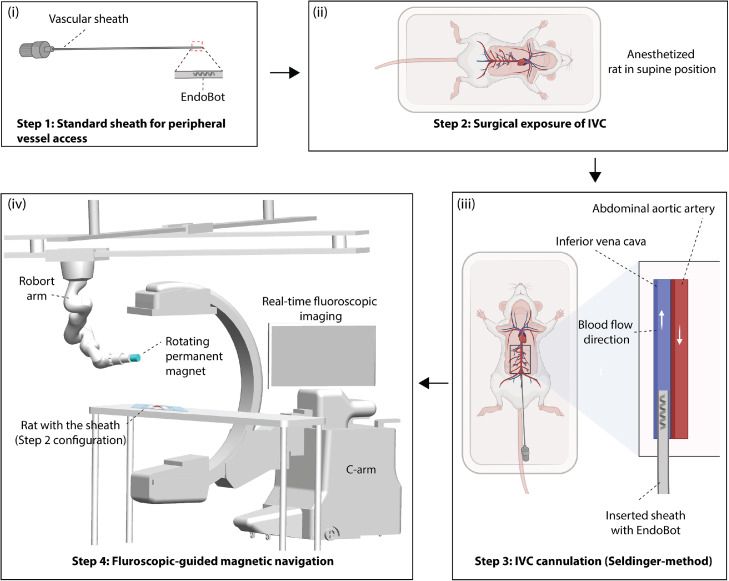
**Experimental workflow for in vivo evaluation of untethered endovascular microrobots in a non-survival rat model.** (i) Standardized peripheral vascular access with deployment of a model, physiologically compatible endovascular microrobot (EndoBot)[4]. (ii) Surgical exposure of IVC. (iii) IVC cannulation using a Seldinger-based technique. (iv) Fluoroscopy-guided magnetic navigation using a robot-arm-mounted magnet integrated with X-ray imaging.

The overall experimental workflow is illustrated in Fig. 1. Briefly, the procedure consists of surgical exposure of the IVC, followed by catheter-based deployment of the microrobot and fluoroscopic-guided navigation under magnetic actuation. The platform integrates vascular access, physiologically relevant flow conditions, real-time imaging, and external magnetic control within a controlled in vivo environment. The following sections describe each step of the protocol in detail.

### Animal preparation and vessel exposure

Adult male Sprague-Dawley rats (300–375 g) were used in non-survival experiments conducted under an approved institutional animal care and use protocol (IACUC A00007480–24). Rat IVC is a vein, as a compliant venous structure, exhibits a variable lumen diameter (1.5–3.3 mm) depending on animal size, anesthetic state, and measurement modality [4,[22], [23], [24]], with peak blood flow velocity ranging from 6 to 10 cm/s [25].

Rats were anesthetized by inhalation of 3 % isoflurane in oxygen (0.75 L/min) for anesthesia induction, followed by intraperitoneal administration of pentobarbital sodium (Leucadia; 50 mg/mL stock solution; 50 mg/kg, IP). Anesthesia was maintained with supplemental pentobarbital doses (20 mg/kg, IP) administered every 50–60 min as needed. Preoperative analgesia was provided via subcutaneous injection of buprenorphine hydrochloride (0.3 mg/mL stock solution; 0.1 mg/kg) [26].

**Fig. 2:**
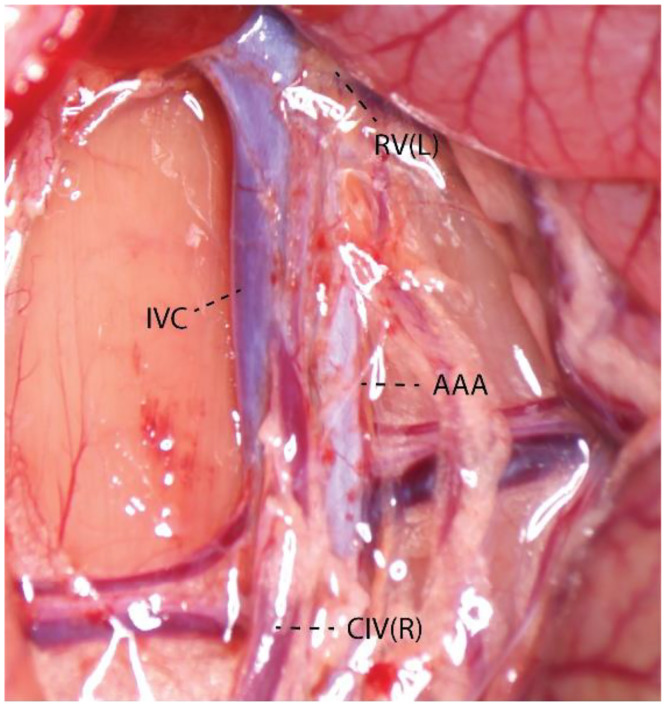
**Anatomical structure around IVC.** RV(L)-Left Renal Vein; AAA-Abdominal Aortic Artery; IVC-Inferior Vena Cava; CIV(*R*)-Right Common Iliac Vein.

After confirmation of deep surgical anesthesia, the rat was positioned supine with limbs secured, and the abdomen was shaved, disinfected, and sterilely draped while normothermia was maintained with a heating pad. A ∼3 cm midline laparotomy was performed to enter the peritoneal cavity, and the small intestine and cecum were gently exteriorized onto prewarmed, saline-moistened gauze to maintain hydration and minimize tissue injury. The infrarenal IVC, identified to the right of the pulsatile abdominal aorta by its darker color and thinner, collapsible wall, was selected between the renal veins and iliac bifurcation (Fig. 2), favoring a distal, relatively branch-free segment for exposure.

### Heparinization

To minimize endovascular coagulation during vessel manipulation, catheterization, and endovascular device deployment, heparinization was performed following surgical exposure of IVC.

### Systemic heparinization

Prior to vascular cannulation, heparin (200 U/kg) was administered endovascularly via the exposed IVC. An adequate circulation time of approximately 1–2 min was allowed to ensure effective anticoagulant distribution before further vascular manipulation.

### Catheter heparinization

All catheters, sheaths and dilators were flushed with heparinized saline (10–20 U/mL) before and during the procedure to prevent clot formation.

### IVC cannulation and endobot introduction

**Fig. 3:**
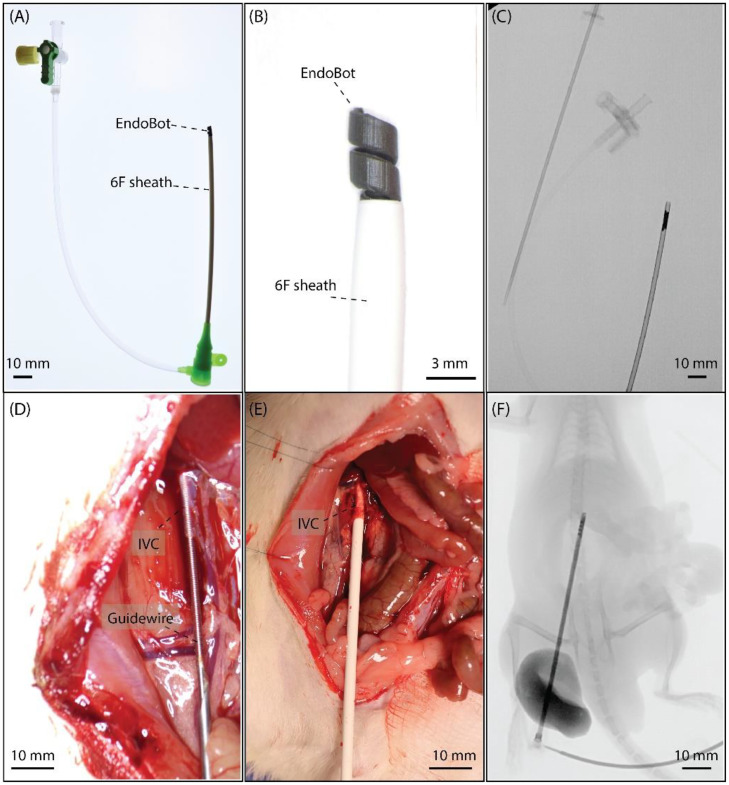
**IVC Cannulation and EndoBot Introduction.** (A) A 6F introducer sheath (Terumo, Tokyo, Japan) preloaded with the EndoBot (EB2.1) at its distal tip. (B) Magnified view of the distal sheath segment showing EB2.1 loaded prior to insertion. (C) Fluoroscopic visualization of the Endobot in the sheath after assembling. (D) A guidewire is inserted into IVC through a needle, needle is withdrawn for better view. (E) Advancement of the 6F sheath into the IVC lumen following venotomy. (F) Fluoroscopic confirmation of sheath positioning within the IVC, with the EndoBot located at the distal sheath tip.

IVC cannulation was performed using a slightly adapted Seldinger technique [27]. A small longitudinal venotomy (∼2.5–3 mm) was made along the guidewire at the puncture site to facilitate sheath insertion. A 6F introducer sheath, preloaded with EndoBot with anas-fabricated diameter of 2.1 mm (EB2.1) at its distal tip (Fig. 3A–C) and introduced without the use of a dilator, was then slowly advanced over the guidewire into the IVC. The sheath was advanced until approximately 1–1.5 cm of its distal segment was positioned within the IVC lumen (Fig. 3D, E). After correct sheath positioning, the guidewire was removed, and the sheath was secured with sterile tape to prevent displacement. At this stage, the EndoBot was located at the distal end of the 6F sheath within the IVC lumen (Fig. 3F), ready for subsequent magnetic actuation.

### Microrobot deployment and retrieval

Upon successful placement of the vascular sheath, the rat was positioned beneath a C-arm fluoroscopic imaging system (OEC 3D, General Electric, Boston, MA). The X-ray detector distance was set at approximately 32 cm to optimize field of view and image quality. An angiogram was then performed using pulse mode and a heparinized contrast agent injected at 5 mL/min for approximately 32 s to delineate the course and anatomy of IVC. Following vascular visualization, magnetic actuation was initiated. After magnetic alignment at the distal tip of the sheath, the EndoBot was carefully released into the IVC. Under continuous fluoroscopic guidance and real-time modulation of the external magnetic field, the Endobot was navigated forward and backward for 2–5 times and subsequently returned to the access site[4]. With the sheath maintained in a fixed position, magnetic actuation was used to guide the EndoBot into the sheath lumen for recapture, and after complete re-sheathing, the sheath-EndoBot assembly was withdrawn together as a single unit.

### IVC tissue harvest and fixation

At the completion of the experimental procedure, IVC was carefully dissected from surrounding tissue and adjacent structures. The IVC segment of interest was gently flushed with saline to remove residual blood and immediately fixed in 10 % neutral buffered formalin (approximately 4 % formaldehyde) solution for subsequent histological and morphometric analyses.

### Actuation and control framework

Magnetic actuation within the testbed utilized an externally applied rotating magnetic field (RMF) with amplitudes ranging from 20 to 50 mT (GM1-ST DC Gauss-meter, AlphaLab, Inc., Salt Lake City, UT, USA) and actuation frequencies between 1 and 30 Hz, consistent with propulsion regimes previously reported for magnetic micro- and millirobots in viscous biological environments [4]. The chosen amplitude range supports torque-dominated locomotion and remains compatible with small-animal in vivo experimentation.

Magnetic propulsion is generated by the torque exerted on a magnetized body, described by ***τ*** = ***m*** × ***B***, where ***m*** represents the magnetic dipole moment and ***B*** the applied magnetic field vector. Effective locomotion requires sufficient magnetic torque to overcome hydrodynamic drag and wall friction under venous flow. Since torque magnitude depends on field strength and relative orientation, the applied field was aligned with the vessel axis to maximize effective torque about the intended rotation axis.

In this study, EndoBot EB2.1 is composed of NdFeB microparticles (∼5 μm) with magnetic properties corresponding to the B-H curve of NdFeB D50 (MQP-15–7–20,065, Magnequench, Germany), yielding an effective ***m*** of approximately 0.46 × 10⁻³ A·m². The device was magnetized using a magnetic yoke as previously described [4].

The actuator-target separation, defined as the distance from the magnet center to the vessel centerline, was maintained between 75 and 100 mm for a 50 × 50 mm cylindrical permanent magnet. These parameters define the torque-generating field experienced by the device and therefore directly govern locomotion performance under the present experimental conditions.

### Imaging setup

All procedures were performed under real-time clinical fluoroscopic imaging using a C-arm system (OEC 3D C-arm, GE Healthcare) operated in cine mode (acquisition rate: 15-30 Hz) to visualize sheath placement, microrobot deployment, and endovascular navigation within the IVC. Sheath positioning and microrobot location were confirmed under fluoroscopy (Fig. 3C, F). Fluoroscopic guidance was also used to verify coaxial alignment between the sheath and the IVC lumen, which is critical for minimizing mechanical resistance and enabling reliable deployment and retrieval.

A brief contrast-enhanced angiographic acquisition was obtained immediately prior to microrobot release to delineate the vessel structure and establish an anatomical reference for image-guided navigation. Contrast administration was limited to short, as-needed acquisitions to minimize perturbation of native hemodynamics and to reduce overall contrast exposure.

Pre-procedure cross-sectional imaging, such as MRI or CT, may be valuable for patient-specific planning in translational or disease-model studies. However, such imaging was not required for this early-stage rat IVC testbed. The objective of this model is to control benchmarking of magnetic actuation and navigation under standardized vascular access conditions, rather than patient-specific anatomical reconstruction. In this context, fluoroscopic confirmation combined with pre-deployment angiography provided sufficient geometric definition for reproducible endovascular testing.

Imaging geometry was maintained consistently throughout each experiment to ensure reliable interpretation of microrobot trajectory. Because magnetic propulsion depends on the spatial relationship between the applied magnetic field vector and the vessel axis, preserving alignment between the field source and vascular geometry was essential to avoid geometric confounders. In addition, because the microrobot was designed to be visible under X-ray, navigation control relied on the observed microrobot position together with the vessel orientation defined from angiographic reference, enabling field alignment that maximizes the effective torque about the intended locomotion axis during propulsion.

Although fluoroscopy was selected for its direct relevance to endovascular practice, the framework is compatible with alternative imaging modalities. High-frequency ultrasound enables real-time vessel diameter and Doppler-based flow assessment [23]. The selection of imaging modality should consider compatibility with magnetic field generation hardware and the intended translational pathway.

For reproducibility, explicit reporting of the imaging modality, angiographic protocol, sheath positioning strategy, and the relative spatial alignment between the field source and the vessel axis is recommended. Clear documentation of these geometric conditions ensures that propulsion performance can be interpreted independently of imaging-related variability.

**Fig. 4:**
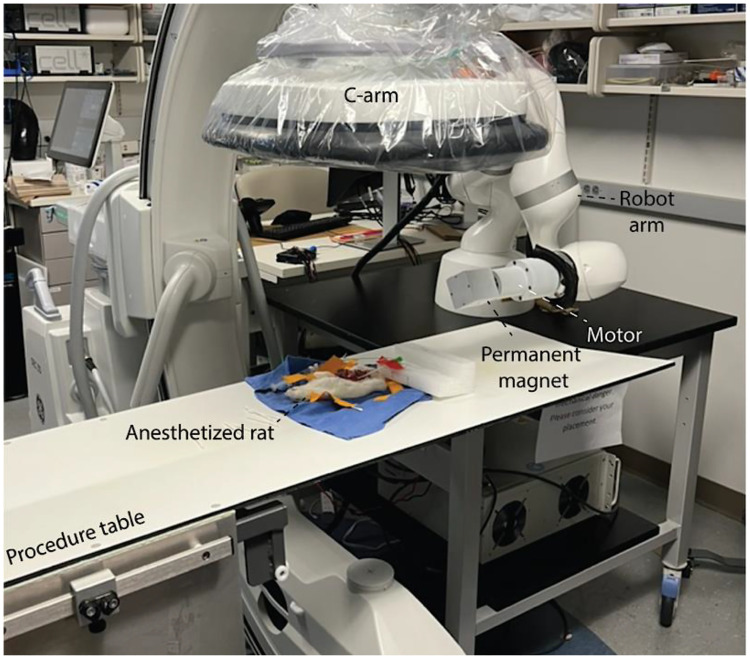
**Experimental configuration of the in vivo magnetic actuation testbed.** The integrated setup combining a C-arm fluoroscope, a robot arm-mounted permanent magnet, and an anesthetized rat positioned on the procedure table. The configuration illustrates the spatial integration of imaging and magnetic actuation hardware during in vivo testing.

Fig. 4 illustrates the in vivo experimental configuration of the fluoroscopic guided magnetic actuation setup. C-arm system uses the same workspace with a permanent magnet mounted on a motor to generate a rotating magnetic field. The magnet assembly is positioned using a robotic arm, enabling precise control over its spatial position and orientation and thereby allowing programmable delivery of magnetic torque.

### Performance metrics for early-stage in vivo evaluation

Deployment feasibility was determined by the successful controlled release of the microrobot from the sheath into the IVC without passive migration by venous flow. Retrieval feasibility can be defined as successful magnetic re-entry into the sheath and complete extraction without device fragmentation or the use of a tethered rescue tools. Both deployment and retrieval performance are challenged by continuous blood flow toward the heart.

**Fig. 5:**
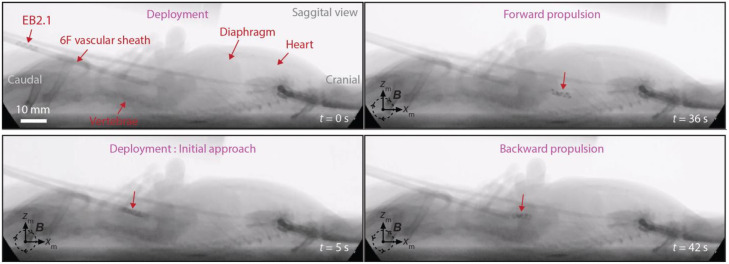
**Fluoroscopic guided navigation demonstration.** Sequential snapshots depict the navigation of EB2.1 performing forward and backward propulsion with rotating magnetic field actuation along the IVC of rat.

Navigation performance was characterized by the ability to execute controlled forward (with flow) and backward (against flow) passes along a predefined IVC segment under continuous blood flow. From the entry point to the the diaphragm, there is approximately 60 mm of IVC segment where the microrobot can be moved. Sequential fluoroscopic snapshots in Fig. 5 demonstrate an EB2.1 advancing cranially (forward propulsion, upper right panel) and subsequently reversing direction (backward propulsion, lower right panel) under rotating magnetic field actuation. Traversal time was recorded for each directional pass (e.g., *t* = 36 s for forward propulsion and *t* = 42 s for backward propulsion in the representative sequence), providing a quantitative measure of propulsion efficiency under access-modified venous flow. Standard pulsed fluoroscopic images also showed the clear visibility of EndoBot in the animal body.

Directional stability was assessed by observing maintenance of longitudinal alignment with the vessel axis during locomotion. In Fig. 5, the microrobot remains visually aligned with the IVC centerline without uncontrolled rotation or lateral drift toward the vessel wall. Sustained alignment under continuous flow served as a qualitative indicator of stable magnetic torque generation and adequate wall interaction.

Together, these image-based observations provide reproducible benchmarks for deployment control, bidirectional navigation, propulsion efficiency, and directional stability within a defined in vivo boundary condition.

Acute procedural safety was evaluated at both gross and histological levels. Gross inspection of the IVC at harvest was conducted to identify visible thrombus formation or vessel wall disruption. Histological analysis assessed endothelial integrity, luminal thrombus presence, and structural injury to the vessel wall. These acute endpoints serve as early indicators of device–vessel interaction safety, without addressing long-term remodeling or chronic thrombogenicity.

Device fragmentation was assessed immediately after retrieval. After the microrobot was re-captured into the sheath and removed from the vasculature, it was visually inspected and compared to its pre-deployment appearance to confirm preservation of overall geometry and the absence of structural breakage or missing segments.

Imaging-based detectability was included as a procedural performance parameter. The microrobot remained visually trackable throughout deployment, navigation, and retrieval under X-ray imaging. Loss of detectability was recorded if the contrast-to-noise ratio decreased below reliable visualization thresholds. Sustained visibility is essential for translational relevance in image-guided endovascular applications.

This performance framework establishes an intermediate validation stage between in vitro testing and survival or large-animal studies. By isolating acute navigational control, mechanical compatibility, and short-term vascular safety under standardized access conditions, the model enables systematic comparison of device designs and actuation strategies prior to translational escalation. Looking ahead, forthcoming survival studies will build upon these acute benchmarks to evaluate long-term device retention, chronic vascular response, and sustained functional performance, thereby completing the continuum toward comprehensive translational assessment.

While the present study demonstrates the framework using an untethered magnetic microrobot, the core methodological elements of the testbed, including sheath-based vascular access, controlled flow conditions, and real-time image-guided evaluation, are agnostic to the actuation modality. As such, the framework can be extended to other wireless microrobotic platforms and propulsion strategies, such as ultrasound-based actuation. Importantly, such adaptations require that the integration of actuation and imaging systems does not compromise procedural access, imaging compatibility, or operational safety, necessitating careful evaluation of these parameters during implementation.

## Failure modes and troubleshooting

### IVC thrombosis

Thrombosis within IVC may occur when substantial luminal occupation and reduced venous flow promote local blood stasis, and when repeated device–wall contact further perturbs the endothelium. This risk may be more pronounced in small-animal models because the sheath–device system occupies a relatively large fraction of the venous lumen and venous shear conditions are lower than in arterial circulation. Prolonged endovascular dwell time can further increase susceptibility to clot formation. During early protocol optimization, thrombotic tendencies were observed in the absence of standardized anticoagulation. After implementation of systemic heparinization, no gross IVC thrombosis was observed during at least 3 h of sheath residence, based on maintained sheath patency and absence of visible clot upon inspection. To mitigate thrombotic risk, we recommend systemic anticoagulation, minimizing unnecessary endovascular manipulation, and gentle intermittent flushing to reduce stagnant flow while avoiding excessive endovascular volume loading.

### Catheter (Sheath) thrombosis

Catheter thrombosis resulted from inadequate flushing, leading to residual blood within the catheter lumen and subsequent coagulation. This complication was observed in at least two animals during initial methodological development without systemic heparinization. Intraluminal clot formation obstructed microrobot deployment and contrast injection, and posed a risk of embolization if flushed into the circulation.

To prevent intrasheath clot formation, the catheter should be immersed in a container with heparin/saline solution for at least 30 min before insertion into the vessel, gently flushed with heparinized saline prior to insertion and periodically throughout the procedure within the total injection limits. Maintaining a continuous saline column within the catheter system preserves patency and reduces embolic risk.

### Deployment failure

Deployment failure, independent of catheter or sheath thrombosis, may arise from geometric and mechanical factors encountered during device loading and advancement. A primary contributor is misalignment between the vascular sheath and the longitudinal axis of the IVC, which increases resistance at the sheath tip and impedes smooth device release. Given the deep anatomical positioning of the IVC within the abdominal cavity, achieving perfect coaxial alignment can be technically challenging. As a result, an angular deviation may exist between the sheath trajectory and the IVC axis. As this angle increases, the sheath tip may approach the vessel wall asymmetrically, reducing the effective clearance for microrobot exit. Excessive advancement of the sheath under such angular misalignment can further exacerbate deployment difficulty. If the sheath tip abuts or partially contacts the venous wall, the microrobot may encounter increased resistance upon release, with limited space to transition smoothly from the sheath lumen into the IVC. In extreme cases, the robot may experience compression or deflection at the tip, preventing controlled advancement into the vessel. In addition, partial collapse of the IVC may occur when local endovascular flow is insufficient to maintain luminal patency around the sheath tip. Unlike arteries, veins are highly compliant structures and do not maintain a fixed circular geometry under reduced flow or external compression. As a result, even minor reductions in venous return can lead to transient narrowing, increasing frictional resistance during device advancement.

Excessive friction between the soft microrobot and the inner wall of the sheath may further compromise deployment, particularly if the device geometry is distorted during loading. Careful and controlled assembly of the device into the sheath as well as mockup in vitro trials is therefore essential to understand and eliminate microrobot-sheath interactions.

To mitigate deployment failure, imaging confirmation of coaxial alignment between the sheath and the IVC can be performed prior to release. When luminal narrowing is suspected, a gentle saline bolus may be administered to restore venous distension and reduce resistance at the sheath tip. During both deployment and retrieval, we connect the sheath to a syringe pump to generate a low, continuous physiologic saline flow at the sheath tip (approximately 6–10 mL/min), which helps maintain luminal openness and reduces stagnation. These measures collectively reduce frictional resistance and facilitate controlled EndoBot release. Maintaining a controlled injection rate minimizes abrupt luminal expansion and reduces deformation- or flow-induced instabilities.

### Microrobot migration and loss of control

An additional deployment-related risk is unintended cranial migration of the device toward the right atrium in the setting of inadequate magnetic control or excessive forward flow. Because venous return is directed toward the heart, loss of stable wall contact may permit passive transport of the microrobot along the flow stream. In our rat model, the migration distance to the cardiac chambers is short (∼45–55 mm), this risk is amplified. Uncontrolled migration into the right atrium may result in mechanical interaction with intracardiac structures and loss of device control. More importantly, resulted in arrhythmia and cardiac arrest which may lead to animal death.

To reduce migration risk, stable magnetic field alignment, imaging guidance, and gradual initial release from the sheath are essential. Avoidance of abrupt flow augmentation during deployment further limits unintended forward displacement. For example, rapid venographic injection may transiently dilate the vessel and increase forward flow velocity, causing the microrobot to slip cranially. In one case, EndoBot folded non-uniformly near anatomical narrowing such as the diaphragmatic segment, with temporary distortion of its helical geometry [4].

### Retrieval failure

Magnetically guided retrieval is intrinsically more demanding than device release, as controlled re-entry into the confined sheath lumen requires precise coaxial alignment and sustained axial traction. Retrieval failure may result from sheath-IVC misalignment, insufficient magnetic gradient strength, suboptimal field orientation, or excessive friction at the sheath tip, particularly when the sheath inner diameter closely approximates the device diameter.

Initial troubleshooting should focus on image-guided reassessment of sheath alignment and realignment of the sheath with the vessel axis. If resistance persists despite appropriate field optimization, upsizing the sheath (typically 1F larger) may reduce friction at the entry interface. As a rescue maneuver, a vascular snare may be employed to gently guide the EndoBot into the sheath; however, excessive traction should be avoided, as undue force may compromise structural integrity or result in device fragmentation [4].

## Scope, boundary conditions and practical limitations of the rat IVC testbed

### Defined scope of the testbed

This study establishes a standardized, early-stage in vivo rat IVC model for the evaluation of untethered microrobots intended for endovascular applications. The testbed enables assessment of endovascular deployment/retrieval feasibility, magnetic actuation and image-guided navigation, positional stability under continuous blood flow, and acute procedural safety within a living vascular environment.

The model is structured on mechanical and navigational performance rather than therapeutic efficacy. It does not aim to evaluate long-term endothelial remodeling, chronic inflammatory responses, sustained drug-release kinetics, or clinical outcomes. Instead, it provides a controlled in vivo setting in which device-vessel interaction and locomotion behavior can be examined under reproducible geometric and hemodynamic conditions while acknowledging animal-to-animal anatomic variations. All findings should therefore be interpreted within the context of short-term endovascular benchmarking.

### Device-Vessel-Sheath dimensional compatibility

For surface-crawling endovascular robots such as EndoBot, dimensional compatibility between the device and the target vessel is a primary functional determinant. Unlike free-swimming microswimmers or surface microrollers, EndoBot relies on direct endothelial contact for propulsion. Effective locomotion therefore depends on controlled mechanical apposition between the device surface and the vessel wall.

If EndoBot is undersized relative to the lumen, wall contact becomes intermittent, reducing traction and compromising propulsion stability. Conversely, oversizing increases luminal occupation, alters local flow patterns, and may impose unnecessary mechanical stress on the vessel wall. In this context, the relationship between device diameter and vessel diameter is not merely a design preference but a locomotion-dependent requirement.

Smaller free-swimming microrobots operating predominantly within the bloodstream or microrollers that move by spinning on the vessel surface do not require precise diameter matching, as their stability is governed primarily by hydrodynamic forces and propulsion field parameters rather than sustained wall interaction. The dimensional framework described here should therefore be understood as a boundary condition applicable to surface-crawling architectures rather than a universal constraint for all endovascular microrobotic systems.

### Hemodynamic conditions before and after sheath insertion

**Fig. 6:**
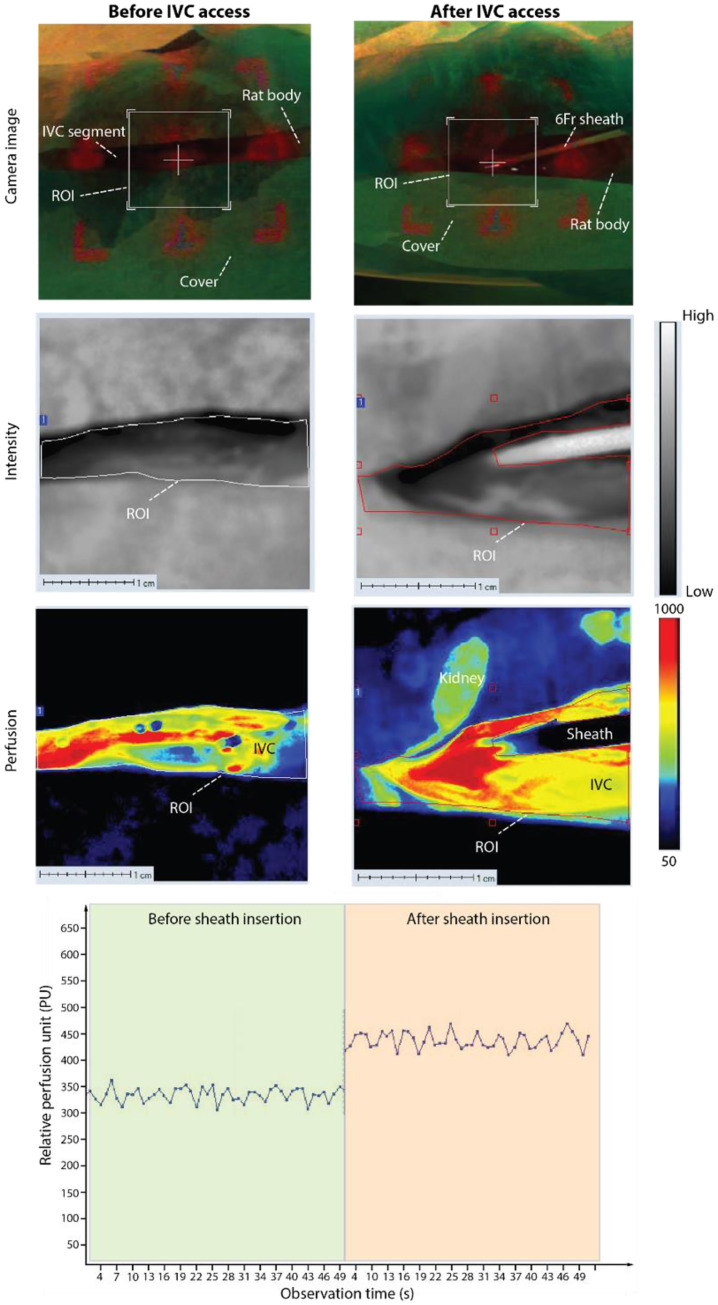
**Sheath-induced alterations in venous hemodynamics in the rat IVC.** Perfusion maps, grayscale intensity images, and real photographs were acquired using laser speckle perfusion imaging before (left) and after (right) IVC access with a 6F sheath. The analyzed region of interest (ROI) is indicated in each panel. Following sheath insertion, perfusion distribution and the PU trace change relative to baseline, consistent with altered local flow conditions. Sheath placement establishes a reproducible, clinically relevant flow condition for evaluating microrobot performance.

Access-induced hemodynamic alterations were investigated using laser speckle perfusion imaging before and after placement of a 6F sheath within the rat IVC. Fig. 6 presents representative perfusion maps, grayscale images, and corresponding perfusion unit (PU) traces. Following sheath insertion, redistribution of the perfusion signal within the region of interest and a consistent upward shift in the PU trace relative to baseline were observed. Because laser speckle imaging provides a relative index of flow rather than absolute velocity, the observed increase from approximately 300–350 PU to 400–450 PU is interpreted as a reproducible modification of local flow conditions resulting from partial lumen occupation and altered flow distribution around the sheath.

This access-induced shift can then be referenced against the reported rat IVC peak velocity range (6–10 cm/s) to ensure that microrobot geometry and magnetic actuation parameters provide sufficient propulsion margin and wall-contact stability under access-modified conditions.

### Use of a structurally normal vessel as a controlled baseline

All experiments were performed in the IVCs of healthy rats. The use of healthy vessels was intentional and aligned with the primary objective of this testbed: to evaluate deployment mechanics, magnetic actuation, and locomotion stability under controlled and reproducible conditions.

Pathological vascular models, such as stenotic, calcified, inflamed, or compliance-altered vessels, introduce additional mechanical and biological variability that can confound interpretation of core system performance. Luminal irregularities, altered wall stiffness, endothelial dysfunction, and inflammatory changes may significantly affect propulsion dynamics, wall interaction, and flow-mediated stability. While such models are highly relevant for disease-specific validation, they introduce layers of complexity that are not necessary at this early-stage system evaluation phase.

By using a geometrically stable and physiologically intact vessel, this testbed isolates the fundamental mechanical and navigational behavior of the microrobotic platform without superimposed pathological variability. This approach enables clearer attribution of performance outcomes to device design and actuation parameters rather than to uncontrolled vascular pathology.

Validation under disease-relevant vascular conditions represents a subsequent translational step to be pursued after baseline system robustness is established in a controlled environment.

### Magnetic actuation constraints

In vascular environments, magnetic torque interaction is significantly modulated by geometry. Vessel curvature, anatomical orientation relative to the applied field, and local confinement alter how the global magnetic field is resolved at the device location. These factors directly influence effective torque transmission, wall contact mechanics, and directional stability. As a result, small deviations in field orientation can lead to reduced control authority or complete loss of propulsion, particularly in tortuous or branching vasculature.

Given this sensitivity, reporting magnetic actuation conditions is essential for reproducibility. Key parameters include field strength, rotation frequency of the external magnet, spatial orientation relative to the vessel axis, actuator-target distance, and control strategy (e.g., open-loop versus closed-loop).

Magnetic actuation introduces safety considerations related to field-device interactions and system-level integration. The field strengths used here (∼20–50 mT at the target) are substantially lower than those encountered in clinical MRI; however, potential risks remain. In worst-case scenarios, unintended interactions may occur with implanted electronic devices or through mechanical effects such as torque or displacement of ferromagnetic components.

The rat IVC model described herein supports both permanent-magnet-based systems and electromagnetic coil setups. In permanent-magnet actuation, a mechanically rotated magnet produces a spatially decaying magnetic field, with magnitude at the vessel location determined by magnet geometry, magnetization, and effective stand-off distance. Permanent magnets provide stable field generation without resistive heating and allow energy-efficient continuous operation with minimal hardware complexity. However, field magnitude and gradient are intrinsically linked to spatial positioning and geometry, which limits independent programmability of field amplitude and direction. Accurate documentation of magnet configuration and stand-off distance is essential for reproducibility.

Electromagnetic coil systems generate rotating fields via controlled current modulation [28], enabling programmable adjustment of field amplitude and orientation within a defined workspace. This flexibility supports advanced vector synthesis and dynamic control strategies. However, sustained operation at higher millitesla levels can result in resistive heating, increased power consumption, and greater hardware complexity. The selection between permanent and electromagnetic actuation therefore involves a trade-off between geometric simplicity and control flexibility.

Permanent magnets cannot be switched off and therefore require precise control of position, orientation, exposure duration, and operating environment. In contrast, electromagnetic coil systems enable dynamic field modulation and more precise rotational control, but introduce additional constraints related to power consumption, heating, and time-varying field exposure. Risk mitigation strategies include maintaining appropriate actuator-target distance, limiting exposure duration, excluding unsecured ferromagnetic objects from the workspace, and adhering to established safety limits. When integrated with robotic manipulators, additional safeguards such as constrained workspace operation, motion planning, and collision avoidance are required.

No interference with fluoroscopic (X-ray) imaging was observed under the experimental conditions used in this study. C-arm systems with flat-panel detectors showed stable imaging performance at the applied field levels, whereas older image-intensifier-based systems may be more susceptible due to electron beam deflection.

To facilitate navigation, short contrast-enhanced fluoroscopic acquisitions can be used to delineate vessel geometry prior to deployment. However, contrast administration should be minimized, even in non-survival models, to avoid unnecessary physiological perturbations that may alter flow conditions and influence locomotion behavior.

### Anticoagulation and acute thrombosis control

Systemic heparinization was administered to minimize the risk of acute thrombosis during vascular cannulation and intraluminal device navigation. Because thrombus formation can significantly influence microrobot mobility, propulsion resistance, and overall procedural success, variability in anticoagulation represents a critical experimental parameter. Insufficient anticoagulation may increase frictional resistance or lead to device entrapment, whereas excessive dosing may introduce bleeding-related confounders. For this reason, the anticoagulation regimen should be clearly specified, including the heparin dose (U/kg), timing relative to cannulation, and any observed thrombotic or bleeding complications. It is important to note that this acute, non-survival model is not designed to assess chronic thrombogenicity, long-term hemocompatibility, or delayed endothelial responses, but rather to ensure stable short-term endovascular conditions during system-level performance evaluation.

### Translational considerations: comparison of rat IVC and human endovascular environments

Early clinical translation of endovascular microrobotic systems is most likely to occur in superficial or readily accessible vascular territories, such as peripheral vessels or arteriovenous access circuits [4]. In these settings, actuator-target separation is minimized, enabling more efficient magnetic field delivery and more reliable image-guided operations. Notably, these conditions partially overlap with those achieved in the rat IVC testbed, including relatively shallow deployment depth, sheath-based access, and physiologically relevant flow conditions. As such, the rat IVC model provides a relevant intermediate platform for evaluating microrobot navigation, actuation, and imaging under conditions that approximate early-stage human applications.

At the same time, the relevance of the rat IVC testbed is not limited to superficial vascular targets. Several core features of the model, such as vessel dimensions, intravascular flow, device-wall interactions, and image-guided navigation, capture fundamental aspects of endovascular operation that extend across both venous and arterial systems (Table 1). In this context, the testbed enables controlled evaluation of key physical and control principles that are directly applicable to more complex human-scale environments.

**Table 1:** Comparison of rat IVC testbed and human-scale vasculature with translational implications.

Parameter	Rat IVC Testbed	Human-Scale Vasculature	Translational Implication of IVC Testbed Validation
**Vessel diameter**	1.5–3.3 mm	Arteries: Veins: 5–30 mm	Validates device operation in small, human-relevant vascular dimensions
**Depth from surface**	∼5–10 mm	∼2–10+ cm (anatomy-dependent)	Provides a practical in vivo imaging and actuation environment for early translational testing
**Vascular geometry**	Relatively straight segment with minimal branching	Long, tortuous vessels with complex branching networks	Establishes baseline navigation performance in a controlled vascular setting
**Flow conditions**	Moderate, continuous venous flow (∼6–10 cm/s)	Pulsatile (arterial) and continuous (venous) flow with higher variability	Enables evaluation under physiologically relevant venous flow conditions
**Access modality**	Sheath-based access	Sheath- and catheter-based clinical access	Confirms procedural compatibility with sheath-based endovascular workflows
**Navigation distance**	Short segments (∼5–7 cm)	Longer paths with cumulative tortuosity and branching	Supports validation of sustained endovascular navigation
**Imaging and actuation constraints**	Superficial anatomy enables high signal-to-noise imaging and efficient magnetic field delivery	Greater depth and clinical constraints limit imaging quality and deliverable actuation fields	Provides an integrated platform to evaluate the feasibility of image-guided operation prior to human-scale implementation

In the present study, the rat IVC depth from the surface of the body was approximately 1 cm; therefore, delivering 20–50 mT magnetic fields was trivial. Under these conditions, stable actuation, controlled navigation, and consistent imaging were achieved within a physiologically relevant venous flow environment[4].

In contrast, human-scale applications involve increased tissue depth (∼2–10+ cm depending on anatomy), greater actuator-target separation, and more complex vascular environments. These include longer navigation distances, vessel tortuosity, hierarchical branching networks, and flow variability driven by cardiac and respiratory dynamics. Because magnetic field strength and gradients decay rapidly with distance, increased actuator-target separation reduces the effective force and torque acting on the microrobot. As a result, maintaining consistent propulsion, controlled wall contact, and precise steering becomes more challenging, potentially leading to off-axis motion, drift, or reduced propulsion efficiency. These challenges are not specific to a single vascular territory but are expected across a range of clinically relevant applications. Additionally, pulsatile flow and vessel motion introduce time-varying perturbations that may affect microrobot stability, magnetic alignment, and device-vessel interactions.

Accordingly, translation to human-scale systems may require improvements in actuation, control, and imaging strategies. These include more efficient field focusing, improved microrobot magnetic properties, enhanced steering precision, and the integration of closed-loop feedback and navigation algorithms to accommodate anatomical and physiological variability.

From an imaging standpoint, increased depth and tissue attenuation can reduce visualization quality compared to the rat model. However, as demonstrated in our previous EndoBot study, the device remained fluoroscopically visible in human cadavers, supporting the feasibility of image-guided translation. Imaging performance may nevertheless vary with anatomy, target depth, and acquisition settings, and should be balanced against radiation exposure in clinical environments. In addition, superficial vascular targets may allow the integration of complementary imaging modalities, such as ultrasound, which are well-suited for real-time guidance in shallow tissues.

## Ethics statements

All procedures were approved by Mayo Clinic Institutional Animal Care and Use Committee (IACUC A00007480–24) and performed in accordance with NIH guidelines.

## Declaration of competing interest

The authors declare that they have no known competing financial interests or personal relationships that could have appeared to influence the work reported in this paper.

## Data Availability

Data will be made available on request.
